# *Escherichia coli aceE* variants coding pyruvate dehydrogenase improve the generation of pyruvate‐derived acetoin

**DOI:** 10.1002/elsc.202200054

**Published:** 2023-01-31

**Authors:** W. Chris Moxley, Rachel E. Brown, Mark A. Eiteman

**Affiliations:** ^1^ Department of Microbiology University of Georgia Athens Georgia USA; ^2^ School of Chemical Materials and Biomedical Engineering University of Georgia Athens Georgia USA

**Keywords:** acetoin, acetolactate synthase, point mutation, pyruvate dehydrogenase, repeated batch fermentation

## Abstract

Several chromosomally expressed AceE variants were constructed in *Escherichia coli ΔldhA ΔpoxB ΔppsA* and compared using glucose as the sole carbon source. These variants were examined in shake flask cultures for growth rate, pyruvate accumulation, and acetoin production via heterologous expression of the *budA* and *budB* genes from *Enterobacter cloacae ssp. dissolvens*. The best acetoin‐producing strains were subsequently studied in controlled batch culture at the one‐liter scale. PDH variant strains attained up to four‐fold greater acetoin than the strain expressing the wild‐type PDH. In a repeated batch process, the H106V PDH variant strain attained over 43 g/L of pyruvate‐derived products, acetoin (38.5 g/L) and 2R,3R‐butanediol (5.0 g/L), corresponding to an effective concentration of 59 g/L considering the dilution. The acetoin yield from glucose was 0.29 g/g with a volumetric productivity of 0.9 g/L·h (0.34 g/g and 1.0 g/L·h total products). The results demonstrate a new tool in pathway engineering, the modification of a key metabolic enzyme to improve the formation of a product via a kinetically slow, introduced pathway. Direct modification of the pathway enzyme offers an alternative to promoter engineering in cases where the promoter is involved in a complex regulatory network.

**Abbreviations**
ALDC: acetolactate decarboxylase
ALS: acetolactate synthase
LB: lysogeny broth
MOPS3‐[N‐morpholino]: propanesulfonic acid
PCR: polymerase chain reaction
PDH: pyruvate dehydrogenase

## 1 INTRODUCTION

Innovative metabolic engineering tools and strategies are essential for building efficient microorganisms to produce biochemicals. Generally, to enhance the formation of a specific product, the pathway to that biochemical is targeted, for example, optimizing enzymes [1, 2, 3] or plasmid constructs [4, 5, 6]. The central goal of these approaches is to modify the expression, regulation, or catalytic activity of enzymes to increase metabolic carbon flux through the pathway leading to the product. Unfortunately, any heterologous pathway competes with native metabolism supporting cell growth. A key branchpoint inevitably exists between native enzymes and the first enzyme in the pathway leading to the product. For example, products derived from pyruvate such as 2,3‐butanediol [7], acetoin [8], and isobutanol [9], must directly compete with several native pathways which could include pyruvate dehydrogenase [10], pyruvate oxidase [11], pyruvate decarboxylase [12], pyruvate carboxylase [13], and lactate dehydrogenase [14].

Many strategies exist to eliminate or reduce competing pathways. For enzymes which are not required for growth, gene knockouts have become routine [15, 16]. More recently, studies have sought to reduce the activity of native pathways by modifying the promoter region of a key enzyme. For example, the native *aceE* promoter was replaced with a modified *vanABK* promoter yielding a strain of *Corynebacterium glutamicum* in which *aceE* expression was controlled by the presence of a P*vanABK*‐sensitive effector molecule [17]. Similarly, CRISPR interference decreases the expression of the pyruvate dehydrogenase complex (PDH) by targeting the promoter regions of *pdhR* and *aceE*, resulting in pyruvate accumulation by *Escherichia coli* [18].

Alternatively, the intrinsic activity of a competing enzyme may be decreased by making substitutions in key residues on that protein in order to reduce but not eliminate flux through the required native pathway [19]. Using this approach, protein variants of AceE, the E1 component of PDH, led to the accumulation of pyruvate in *E. coli* [20]. The use of PDH variants as a metabolic engineering tool could also potentially increase the production of pyruvate‐derived biochemicals.

**FIGURE 1:**
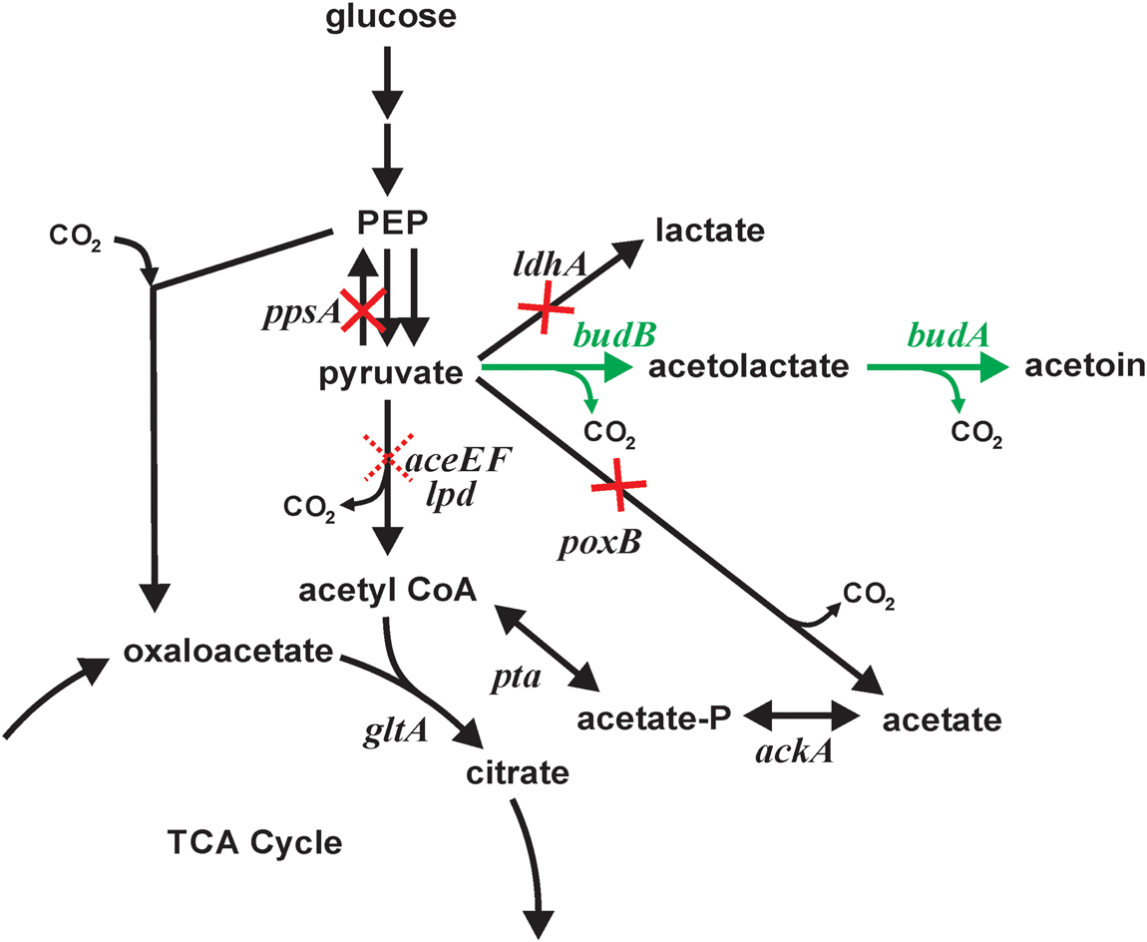
Key biochemical pathways for the conversion of glucose to acetoin in *Escherichia coli*. Gene knockouts performed in this study are indicated by solid red “×”. Flux through the PDH was partly curtailed (dotted red “×”) by the introduction of *aceE* variant alleles coding amino acid substitutions in the E1 component of the complex. Heterologous *budB* and *budA* genes from *Enterobacter cloacae* ssp. *dissolvens* were expressed for the conversion of pyruvate to acetoin (green)

Acetoin, a metabolic precursor to 2,3‐butanediol, is derived from pyruvate (Figure 1) by the enzymes acetolactate synthase (ALS, [21]) and acetolactate decarboxylase (ALDC, [22]). Acetoin is a pale‐yellow liquid with a yogurt odor and butter taste which is commercially used as a flavor or fragrance in foods, cigarettes, cosmetics, and biological pest controls [8, 23]. Acetoin is the simplest acyloin, a compound with a hydroxy group adjacent to a ketone, an important structure useful for the chemical synthesis of various products [8]. Although acetoin is produced via chemical synthesis from fossil feedstocks, interest in microbially produced acetoin is growing, as consumer demand for natural products increases in the cosmetics and food industries [8].

**PRACTICAL APPLICATION**

This contribution provides a strategy to redirect carbon flux from central metabolism towards a product of interest by reducing the activity of a central metabolic enzyme. In this case, the goal is to increase the yield of acetoin from glucose by modifications in the E1 component of the pyruvate dehydrogenase complex in *Escherichia coli*. This approach has practical application toward any product derived from pyruvate, or more generally, by targeting other metabolic enzymes, any product derived from a key branchpoint in metabolism.

Many bacteria and yeast naturally produce acetoin as part of the butanediol fermentation pathway. *E. coli* lacks the complete butanediol fermentation pathway, but generates intermediate acetolactate during valine biosynthesis. One strategy for microbial acetoin production in native butanediol producers is to limit the conversion of acetoin to 2,3‐butanediol. For example, overexpression of a water‐forming NADH oxidase in *Serratia marcescens*, a native 2,3‐butanediol producer, achieved an acetoin titer of 75.2 g/L with a 52% reduction of 2,3‐butanediol titer [24]. This approach to increase acetoin titer has also been successfully applied to other native producers including *Bacillus subtilis* and *Klebsiella pneumoniae* [25, 26]. While there are many native acetoin and 2,3‐butanediol producers, non‐native hosts, such as *E. coli*, have also been engineered to produce these compounds. The general strategy to engineer acetoin producing *E. coli* strains is to express heterologous genes encoding ALS and ALDC from native acetoin or butanediol producing microorganisms. For example, *E. coli* expressing *alsS* (encoding ALS) from *B. subtilis* and *alsD* (encoding ALDC) from *Aeromonas hydrophila* achieved an acetoin titer of 21 g/L [27]. Often paired with heterologous gene expression is the inactivation of by‐product pathways. For example, knockouts in *ldhA*, *pta*, *ackA*, *butA*, and *nagA* decreased by‐product formation and increased acetoin titer five‐fold to 14 g/L in engineered *C. glutamicum* strains [28].

In *E. coli* and many other bacteria, pyruvate is primarily metabolized to acetyl‐CoA by PDH under aerobic conditions. Thus, ALS directly competes for the substrate pyruvate with AceE, the pyruvate‐binding subunit of PDH. Strategies aimed at reducing this competition include deleting *aceE* to direct pyruvate flux toward ALS. For example, 0.87 g/L of acetoin was produced when genes for ALS and ALDC were expressed in an *E*. *coli* strain containing a deletion in *aceEF* [29]. While a deletion of *aceE* can increase the availability of pyruvate for acetoin production, under aerobic conditions this strategy requires the supplementation of an additional carbon source such as acetate [30, 31]. Rather than eliminate the activity of AceE, an alternative strategy would be to reduce the activity of AceE by modification of the amino acid sequence of the enzyme itself. This strategy does not preclude other approaches, such as knockouts in genes coding for competing pathways not required for growth.

The aim of this study was to examine AceE variants as a tool to increase the production of the pyruvate‐derived biochemical acetoin. We hypothesize that reducing the flux through PDH will enable increased acetoin production during growth on defined medium with glucose as the sole carbon source. Alleles encoding AceE variants were engineered into *E. coli* W containing deletions in pyruvate oxidase (*poxB*), lactate dehydrogenase (*ldhA*), and phosphoenolpyruvate (PEP) synthetase (*ppsA*). An acetoin pathway containing ALS (*budB*) and ALDC (*budA*) from *Enterobacter cloacae* ssp. *dissolvens* was expressed in each strain to test the impact of AceE variants on acetoin yield.

## 2 MATERIALS AND METHODS

### 2.1 Media

Cultures were routinely grown on Lysogeny Broth (LB) during plasmid and strain construction, while *aceE* mutants were grown on TYA medium containing (per L) 10 g tryptone, 5 g NaCl, 1 g yeast extract, and 1 g sodium acetate trihydrate [32]. As needed, antibiotics were included in medium (final concentration): ampicillin (100 µg/ml), kanamycin (40 µg/ml), and chloramphenicol (20 µg/ml). For counter‐selection against *sacB*, the medium was supplemented with 100 g/L sucrose, and NaCl was excluded.

The defined basal medium to which carbon/energy sources were added contained (per L): 8 g NH_4_Cl, 1.2 g KH_2_PO_4_, 1.0 K_2_HPO_4_, 2.0 g K_2_SO_4_, 0.6 g MgSO_4_·7H_2_O, 0.25 mg ZnSO_4_·7H_2_O, 0.125 mg CuCl_2_·2H_2_O, 1.25 mg MnSO_4_·H_2_O, 0.875 mg CoCl_2_·6H_2_O, 0.06 mg H_3_BO_3_, 0.25 mg Na_2_MoO_4_·2H_2_O, 5.5 mg FeSO_4_·7H_2_O, 20 mg Na_2_EDTA·2H_2_O, 20 mg citric acid, 20 mg thiamine·HCl. In shake flask cultures used 20.9 g 3‐[*N*‐morpholino]propanesulfonic acid (100 mM MOPS) while for batch processes used 25 mM MOPS. Thiamine was filtered sterilized, and other medium components were autoclaved in compatible mixtures, combined and then adjusted to a pH of 7.1 with 20% (w/v) NaOH.

### 2.2 Strains and genetic modifications

Strains used in this study are shown in Table 1. Gene knockouts in *E. coli* W were constructed by methods previously described [33]. Knockouts were selected on plates supplemented with kanamycin. Forward primers external to the target gene and reverse primers within the kanamycin resistance cassette were used to confirm proper chromosomal integration (Table S1). The kan^R^ marker was removed by expression of FLP recombinase from pCP20 [33]. Gene knockouts and removal of the markers were verified by PCR. To construct MEC1320, the chloramphenicol‐*sacB* (cam‐*sacB*) cassette and 200 bp of homology flanking *aceE* was amplified from purified genomic DNA of MEC813 [20] and integrated into the *aceE* locus of MEC1319 expressing the lambda red system from pKD46. Nucleotide sequences of homologous regions used to integrate DNA into the *aceE* locus were identical in *E. coli* C (ATCC 8739) and *E. coli* W (ATCC 9637).

**TABLE 1:** Strains used in this study

Strain	Relevant characteristics	Reference
ATCC 8739	*Escherichia coli* C	Wild‐type
ATCC 9637	*Escherichia coli* W	Wild‐type
MEC813	ATCC 8739 Δ*ldhA* Δ*poxB* Δ*aceE*::cam‐*sacB*	[20]
MEC1122	ATCC 8739 Δ*ldhA* Δ*poxB* Δ*ppsA* Δ*aceE*::cam‐*sacB*	This study
MEC1319	ATCC 9637 Δ*ldhA* Δ*poxB* Δ*ppsA*	This study
MEC1320	MEC1319 Δ*aceE*::cam‐*sacB*	This study
MEC1321	MEC1319 Δ*aceE*::Kan	This study
MEC1322	MEC1319 Δ*aceE*	This study
MEC1329	MEC1122 Δ*aceE*::*aceE* ^[N276S;R465C;V668A;Y696N]^	This study
MEC1330	MEC1122 Δ*aceE*::*aceE* ^[V169A;P190Q;F532L]^	This study
MEC1332	MEC1319 Δ*aceE*::*aceE* ^[H106V]^	This study
MEC1339	MEC1319 Δ*aceE*::*aceE* ^[N276S;R465C;V668A;Y696N]^	This study
MEC1340	MEC1319 Δ*aceE*::*aceE* ^[V169A;P190Q;F532L]^	This study
MEC1341	MEC1319 Δ*aceE*::*aceE* ^[H106M]^	This study
MEC1342	MEC1319 Δ*aceE*::*aceE* ^[H106M;E401A]^	This study

To construct MEC1329 and MEC1330, error‐prone PCR was used to generate an *aceE* fragment with random mutations and was subsequently integrated into the *aceE* locus of MEC1122 expressing the lambda red system from pKD46. The error‐prone PCR fragment of *aceE* was generated using Stratagene GeneMorph II Random Mutagenesis Kit (Stratagene California, San Diego, CA, USA) using linearized (Spe1) pCM02 as template [20]. PCR was performed according to the manufacturer's specifications. The fragment was gel purified, and 100 ng used to transform MEC1122. Cells were recovered with TYA for 2 h at 30°C. The cells were centrifuged (5,000 × g for 1 min) and washed with 1 ml 0.9% NaCl, then 100 µl of the washed recovery was plated to defined medium agar supplemented with 2.5 g/L glucose and 100 g/L sucrose. Plates were incubated at 30°C for 2–3 days. Positive transformants were verified by PCR and subsequently screened for growth rate and pyruvate accumulation (data not shown). Two strains with decreased growth rates were chosen for sequencing to determine mutations.

Each *aceE* variant allele was PCR amplified from genomic DNA containing the respective allele and integrated into MEC1320 expressing the lambda red system from pKD46. Counter‐selection against *sacB* was used to select mutants that lost the cam‐*sacB* cassette by plating transformants on medium containing sucrose [34]. Colonies were confirmed by colony PCR, and point‐mutated *aceE* genes were amplified from the chromosome, gel purified, and sequenced to confirm mutations.

### 2.3 Plasmid construction

Plasmids used in this study are listed in Table S2. Plasmids were constructed using NEBuilder® HiFi Assembly (New England Biolabs, Ipswich, MA, USA). Phusion® High‐Fidelity Polymerase (New England Biolabs, Ipswich, MA, USA) or PrimeSTAR® Max High‐Fidelity Polymerase (Takara Bio, Mountain View, CA, USA) was used to amplify DNA for cloning and genome integration. Quick‐DNA Miniprep and Zyppy™ Plasmid Miniprep Kits were used to purify genomic and plasmid DNA (Zymo Research, Irvine, CA, USA). DNA Clean and Concentrator, and Zymoclean™ Gel DNA Recovery Kits were used to purify PCR fragments (Zymo Research, Irvine, CA, USA). Restriction enzymes were purchased from New England Biolabs. Plasmids were confirmed by restriction digest and sequencing (ACGT, Inc., Wheeling, IL, USA).

To construct 44_ediss from 445_ediss gifted by Stefan Pflügl [35], primers were used to amplify a linear fragment containing the plasmid backbone, *budA*, and *budB*, then subsequently circularized to create the *budB*‐*budA* operon. The new plasmid 44_ediss was confirmed by restriction digest and sequencing.

### 2.4 Shake flask experiments

A single colony from an LB plate was used to inoculate 3 ml TYA. After 6–10 h of growth, this culture was used to inoculate 3 ml of basal medium with 5 g/L D‐(+)‐glucose to an initial optical density at 600 nm (OD) of 0.05. After 8–12 h of growth, this culture was used to inoculate three 500 ml baffled shake flasks containing 50 ml of basal medium with 5 g/L glucose to an OD of 0.02. All cultures were grown at 37°C on a rotary shaker at 225 rpm. Flasks were sampled for measurement of growth rate and/or extracellular metabolite concentrations. Some flasks were supplemented with 2.34 g/L Na(CH_3_COO)·3H_2_O (1 g/L acetate) as described.

### 2.5 Batch and repeated batch processes

A single colony from an LB plate was used to inoculate 3 ml TYA. After 6–10 h, this culture was used to inoculate a 250 ml shake flask containing 50 ml of basal medium with 20 g/L glucose to an OD of 0.02. When the shake flask culture reached an OD of 1.5‐2, the 50 ml were used to inoculate a 2.5 L bioreactor (Bioflo 2000, New Brunswick Scientific Co., New Brunswick, NJ, USA) containing 1.2 L basal medium with 40 g/L glucose. Duplicate batch processes were performed, and some cultures were supplemented with 18.72 g/L Na(CH_3_COO)·3H_2_O (8 g/L acetate) as described. A repeated batch process started as a batch process described in Section 2.4 except the medium was modified to increase NH_4_Cl from 8 g/L to 10 g/L. Four times, approximately each time glucose was depleted, 56 ml of DI water with 44 g glucose, 40 mg kanamycin, 150 mg KH_2_PO_4_, 125 mg K_2_HPO_4_, 1.25 mg FeSO_4_·7H_2_O and 75 mg MgSO_4_·7H_2_O was added to the fermenter.

Batch studies were conducted with an initial agitation of 400 rpm and at 37°C. Air and/or oxygen‐supplemented air was sparged at 1.25 L/min and agitation was adjusted up to 500 rpm to maintain a dissolved oxygen concentration above 40% of saturation. The pH was controlled at 7.0 using 30% (w/v) KOH or 20% (w/v) H_2_SO_4_. Antifoam 204 (Sigma) was used as necessary to control foaming.

### 2.6 Analytical methods

The optical density at 600 nm (OD) (UV‐650 spectrophotometer, Beckman Instruments, San Jose, CA, USA) was used to monitor cell growth. Samples were routinely frozen at ‐20°C for further analysis, and thawed samples were centrifuged (4°C, 10,000 × g for 10 min), and filtered (0.45 µm nylon, Acrodisc, Pall Corporation, Port Washington, NY). Liquid chromatography using a 7.8 × 300 mm Coregel 64H column (Concise Separations, San Jose, CA, USA) at 60°C with 4 mN H_2_SO_4_ as mobile phase was used to quantify pyruvate, glucose and organic products using RI detection [36]. Students *t*‐test was used to compare data statistically, with 95% confidence interval the basis for significance.

## 3 RESULTS

### 3.1 Variant strain screening for pyruvate and acetoin yield

Pyruvate occupies a key node in central metabolism. Any biochemical product derived from pyruvate in *E. coli* must compete with native pathways including pyruvate dehydrogenase, lactate dehydrogenase and pyruvate oxidase. Although deletions in genes coding for lactate dehydrogenase, pyruvate oxidase and PEP synthetase have minimal effect on aerobic cell growth and metabolism, a knockout in any of the three components of the PDH results in a growth requirement for acetate [31]. An alternative approach to reducing flux through PDH would be to reduce the intrinsic activity of the complex, for example, by amino acid substitutions affecting enzyme activity. Pyruvate accumulation at a yield of 0.66 g/g from glucose has previously been demonstrated in strains expressing variants of AceE, the E1 component of the PDH [20]. The goal of this study was to examine the use of AceE variants for a product derived from pyruvate, acetoin. Our hypothesis was that the increased availability of pyruvate caused by the bottleneck created at PDH would increase the yield of pyruvate‐derived acetoin.

**FIGURE 2:**
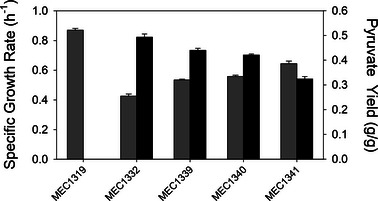
Comparison of *Escherichia coli* W Δ*ldhA* Δ*poxB* Δ*ppsA* AceE variants grown in shake flasks with 5 g/L glucose: specific growth rate (h^−1^, gray bars) and pyruvate yield (g/g, black bars). Error bars indicate standard deviation from three replicates

*E. coli* W containing deletions in the *ldhA*, *poxB*, and *ppsA* genes (MEC1319) was chosen as the host for acetoin production. Several *aceE* variant alleles from *E. coli* C strains [20] were introduced into MEC1319 and screened in triplicate shake flasks for growth rate and pyruvate yield. We also examined MEC1322 which contains a deletion of the *aceE* gene, necessitating the supplementation of acetate into the medium. MEC1319 attained a growth rate of 0.87 ± 0.01 h^−1^, and no pyruvate accumulated. Of the variants able to grow on glucose as the sole carbon source (Figure 2), MEC1332 (AceE[H106V] variant) attained the highest pyruvate yield of 0.49 ± 0.01 g/g while MEC1341 (AceE[H106M]) attained the lowest pyruvate yield of 0.32 ± 0.01 g/g. MEC1339 (AceE[N276S;R465C;V668A;Y696N]) and MEC1340 (AceE[V169A;P190Q;F532L] attained similar pyruvate yields of 0.44 ± 0.01 g/g and 0.42 ± 0.00 g/g, respectively. Pyruvate yield correlated inversely with maximum specific growth rate. MEC1332 attained the lowest growth rate of 0.43 ± 0.01 h^−1^, almost 50% lower than MEC1319 expressing the native AceE. Each measured growth rate and yield was significantly different from other measured growth rates and yields (*p* < 0.05).

MEC1342 (AceE[H106M;E401A]) and MEC1322 (Δ*aceE*) exhibited limited growth on glucose as the sole carbon source (data not shown). Thus, for these two strains only, the medium was supplemented with 1 g/L acetate to support biomass formation. After growth and acetate depletion, MEC1342 and MEC1322 converted glucose to pyruvate at yields of 0.39 ± 0.02 g/g and 0.45 ± 0.02 g/g, respectively.

**FIGURE 3:**
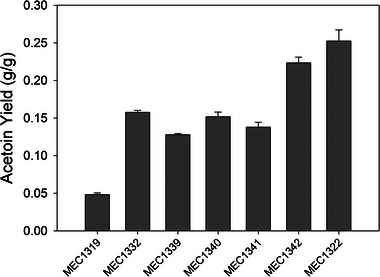
Comparison of *Escherichia coli* W Δ*ldhA* Δ*poxB* Δ*ppsA* AceE variants harboring plasmid 44_ediss grown in shake flasks with 5 g/L glucose: acetoin yield (g/g, gray bars). Error bars indicate standard deviation from three replicates. MEC1322/44_ediss and MEC1342/44_ediss were supplemented with 1 g/L acetate to support growth

In order to understand the impact of each AceE variant on acetoin yield, an acetoin production pathway was introduced into each strain via transformation of the 44_ediss plasmid. The 44_ediss plasmid expresses acetolactate synthase, encoded by the *budB* gene, and acetolactate decarboxylase, encoded by the *budA* gene, each from *E. cloacae* ssp. *dissolvens* (Figure 1). Each transformed strain was examined for acetoin generation in triplicate flask cultures containing 5 g/L glucose or 5 g/L glucose plus 1 g/L acetate (Figure 3). Of the strains that grew on glucose as the sole carbon source, MEC1319/44_ediss displayed the lowest acetoin yield of 0.05 ± 0.00 g/g while MEC1332/44_ediss attained the greatest yield of 0.16 ± 0.00 g/g. MEC1341/44_ediss, MEC1340/44ediss, and MEC1339/44_ediss attained yields of 0.13–0.15 g/g. The difference in acetoin yields of the pairs MEC1339/MEC1341, MEC1341/MEC1340, and MEC1340/MEC1332 were not significantly different (*p* > 0.05). For these strains, high pyruvate yield in the absence of the acetoin pathway plasmid did not predict high acetoin yield when the heterologous pathway was introduced. The two strains with a severe restriction in PDH, which therefore required an acetate supplement, attained the highest acetoin yields. Specifically, MEC1322/44_ediss and MEC1342/44_ediss attained yields of 0.25 ± 0.01 g/g and 0.22 ± 0.01 g/g, respectively, and these yields were significantly different from each other and from yields achieved by other strains (*p* < 0.05).

### 3.2 Controlled batch processes

**FIGURE 4:**
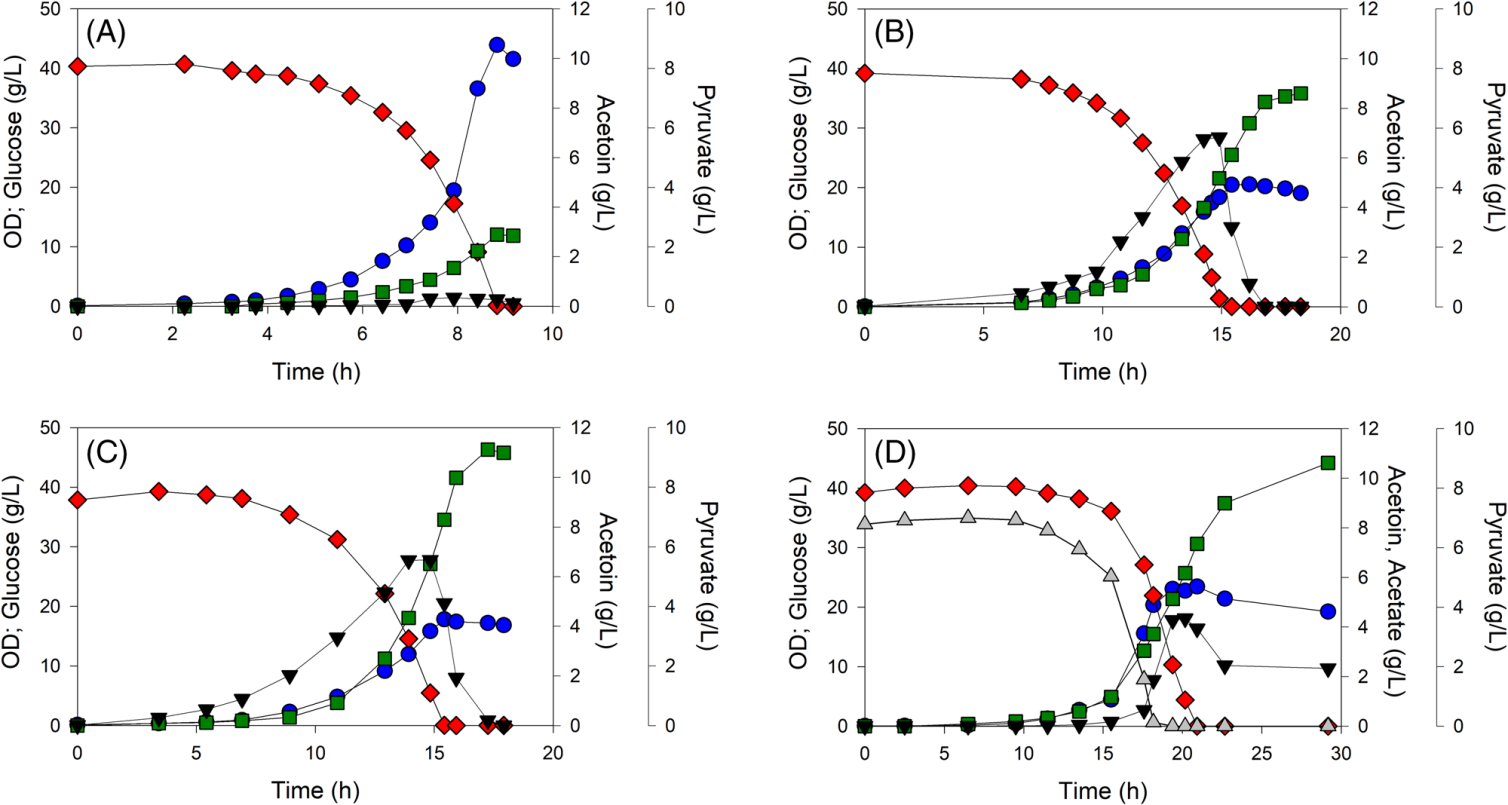
Controlled 1.25 L batch growth of *Escherichia coli* W Δ*ldhA* Δ*poxB* Δ*ppsA* AceE variants harboring plasmid 44_ediss with 40 g/L glucose. (A) MEC1319; (B) MEC1340; (C) MEC1332; (D) MEC1342. Glucose (⧫), pyruvate (▼), OD (⚫), acetate (▲), acetoin (■)

During shake flask screening, acetoin titer was limited by low initial glucose concentration and potentially the absence of pH and dissolved oxygen control. Thus, selected strains were grown in batch culture in controlled bioreactors using 40 g/L glucose (Figure 4). The medium was supplemented with 8 g/L acetate for the growth of MEC1342/44_ediss, which had limited ability to grow on glucose as the sole carbon source. MEC1319/44_ediss generated acetoin at a yield of 0.07 g/g and achieved a final titer of 2.7 g/L (Figure 4A). MEC1340/44_ediss achieved a final acetoin titer of 8.5 g/L at a yield of 0.22 g/g (Figure 4B) while MEC1332/44_ediss achieved a final acetoin titer of 11.2 g/L at a yield of 0.28 g/g (Figure 4C). For both these two variants, the culture accumulated about 6 g/L pyruvate by the time that glucose as depleted, and then the pyruvate was itself consumed with continued generation of acetoin. MEC1342/44_ediss, grown on acetate‐supplemented medium, converted glucose to acetoin at a yield of 0.26 g/g and achieved a final acetoin titer of 10.5 g/L (Figure 4B). However, for this variant, after glucose and acetate were depleted, growth ceased, acetoin production slowed, and nearly 2 g/L pyruvate remained.

Acetolactate accumulated during growth on glucose, reaching a maximum concentration of about 1.5 g/L for MEC1332 and MEC1340 expressing 44_ediss and 2.5 g/L for MEC1342/44_ediss at the time of glucose depletion. Acetolactate had been fully metabolized by the end of each process.

### 3.3 Repeated batch process

**FIGURE 5:**
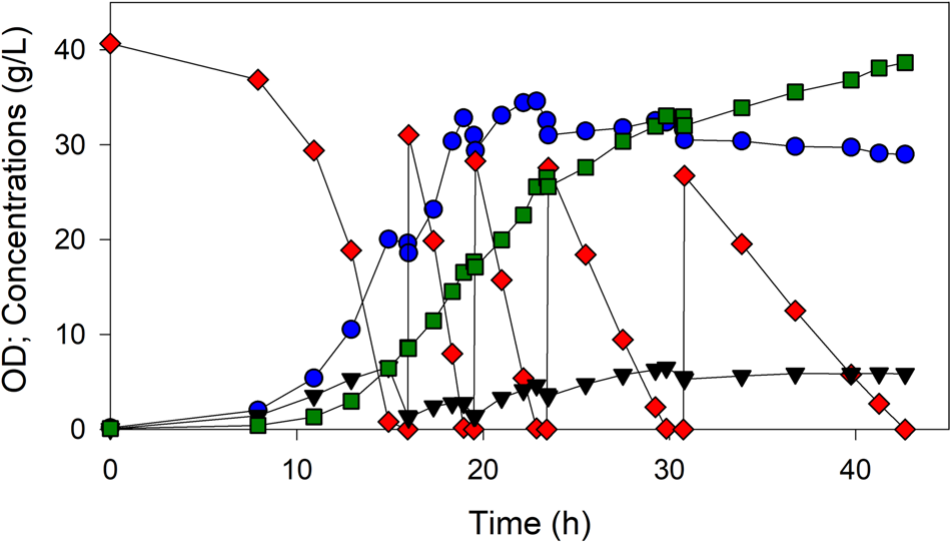
Controlled 1.25 L repeated‐batch growth of MEC1332 (W Δ*ldhA* Δ*poxB* Δ*ppsA* Δ*aceE*::*aceE* ^[H106V]^) harboring plasmid 44_ediss initially with 40 g/L glucose. A 56 ml solution containing 44 g glucose and 40 mg kanamycin was added (four times) when glucose was depleted. Glucose (⧫), pyruvate (▼), OD (⚫), acetoin (■)

Because MEC1332/44_ediss (containing AceE[H106V]) attained the greatest acetoin concentration under batch conditions, this strain was grown in a repeated‐batch culture. In this case, 44 g glucose was added to the culture four times when glucose was depleted. MEC1332/44_ediss achieved an acetoin titer of 38.5 g/L (effectively 52.8 g/L with dilution) with an overall yield of 0.29 g/g and productivity of 0.9 g/L·h (Figure 5). Acetolactate and 2R,3R‐butanediol were also detected during the process. Acetolactate reached a maximum concentration of less than 2 g/L and was fully metabolized by the end of the process. Butanediol was first detected at 17.4 h, and approximately 5 g/L had accumulated by the end of the process.

## 4 DISCUSSION

The goal of this study was to assess the use of *aceE* variants to increase the production of acetoin, a pyruvate‐derived biochemical. The Δ*aceE* strain and five *aceE* variant loci engineered into the chromosome of *E. coli* W were examined for acetoin production.

Of the five strains expressing different *aceE* variants, three (AceE[H106V], AceE[H106M], and AceE[H106M;E401A]) were previously generated using rational protein engineering [20]. Of the residues substituted in these variants, H106 is in the active site cleft and may contribute to orienting pyruvate in the active site, while E401 is positioned on a mobile loop gating the active site and contributes to loop stability [37, 38]. Two novel variants that allow pyruvate accumulation in *E. coli* were additionally generated using a random approach: MEC1339 (AceE[N276S;R465C;V668A;Y696N]) and MEC1340 (AceE[V169A;P190Q;F532L]).

Of the substitutions found in MEC1339, V668A and Y696N are located near the active site channel [37]. An alpha helix proximal to the active site spans residues A663 – G679. Since V668 is positioned in the alpha helix and Y696 is oriented towards the N‐terminal of the alpha helix, these mutations could be destabilizing the alpha helix and the active site channel structure. N276 is oriented near and facing the N‐terminal tail of AceE, proximal to residues 261–263 which form part of the active site cleft [37]. Therefore, N276S could impact the active site structure and/or the position of the N‐terminal tail which interacts with AceF and is important for PDHc activity [39]. R465C is spatially located near a strand of residues 177–186, which contains the active site residue Y177 [37]. R465C could be affecting activity through altering the position of this strand and thus shifting the position of Y177 in the active site.

Of the substitutions found in MEC1340, P190Q is the most radical substitution and is located closest to the active site. P190 is proximal to important active site residues V192 and M194 which provide stabilizing hydrogen bonds to the thiamine diphosphate cofactor. The disruption in bond angles as a result of the P190Q substitution likely destabilizes thiamine diphosphate binding to the active site [37]. F532L is located in an alpha helix and proximal to active site residues D521 and E522, residues predicted to function in substrate channeling between the AceE and the lipoyl group of AceF. Similar to N267S and R465C from MEC1339, this substitution could impact activity by shifting the position of active site residues. V169A is located near the surface the AceE dimer and at the interface between the two AceE monomers, but might not impact activity or structure due to the apparent lack of interactions with nearby residues. Since these two variant strains were generated by random mutagenesis, some of these substitutions may have limited effect or their effect only occurs when the substitutions are made collectively.

Each *aceE* allele was integrated into an *E. coli* W strain containing knockouts in *poxB*, *ldhA*, and *ppsA*, then screened for growth rate and pyruvate yield. The resulting strains exhibited inversely correlated growth rates and pyruvate yields. Each variant allele likely confers unique carbon flux redistribution at the pyruvate node. This effect is also evident when the strains were screened for acetoin production in shake flasks. Strains containing variant *aceE* alleles attained ∼1.6–4‐fold higher acetoin yields than the strain expressing the wild‐type *aceE*.

The variant strains harboring the 44_ediss plasmid differed in their ability to accumulate acetoin. All variants accumulated pyruvate and acetoin concurrently when grown in shake flask and controlled batch culture, and converted residual pyruvate to acetoin after glucose was depleted. The conversion of pyruvate to acetoin requires/generates no ATP nor NAD(P)H, and the generation of CO_2_ serves as the irreversible driving force for acetoin formation. Accumulation of pyruvate itself indicates carbon flux into the acetoin pathway limited acetoin production, and increasing product pathway efficiency may increase acetoin production rate and minimize pyruvate accumulation. Those strains studied which did not require acetate for growth were able to convert the accumulated pyruvate to acetoin after glucose depletion (Figures 4A–C). However, MEC1342/44_ediss, having a severe *aceE* mutation that nearly eliminates growth on glucose as the sole carbon source, was unable to convert all the accumulated pyruvate to acetoin (Figure 4D). MEC1322/44_ediss, lacking *aceE*, generated about the same acetoin as MEC1342/44_ediss in shake flask culture (Figure 3). Thus, an optimal PDH flux for acetoin generation exists: if PDH has (large) wild‐type activity, then a large fraction of glucose is used for native metabolism and acetoin yield is low, while if PDH is severely restricted, then some residual pyruvate remains unconverted, and the acetoin yield is also low. An intermediate PDH flux balances the need for growth and energy with the goal of acetoin formation.

Cells with severe *aceE* mutations growing on pyruvate were unable to provide sufficient energy to meet the maintenance requirements. Previously, AceE variant strains with *ldhA* and *poxB* knockouts were shown to consume accumulated pyruvate to support growth [20], and an additional deletion of *ppsA* was required to block the metabolism of pyruvate. Thus, limiting pyruvate consumption through a restricted PDH did not support growth, suggesting the energy generated from acetyl‐CoA formation and metabolism is insufficient for biomass formation. In the current study, AceE variant strains overexpressing the acetoin pathway in batch culture produced a similar growth phenotype: biomass formation ceased upon glucose depletion (Figure 4). As protein synthesis and turnover can account for 50% of total maintenance energy requirements [40, 41, 42], the continued formation of acetoin in a medium of pyruvate as the sole carbon source (after glucose depletion) appears to be contingent on some PDH flux to generate a threshold level of energy. Energy generated from pyruvate metabolism in a Δ*ldhA* Δ*poxB* Δ*ppsA* strain must come from PDH itself and from the metabolism of acetyl‐CoA. One route for ATP formation is via the Pta‐AckA pathway through acetate formation [43, 44], though intracellular acetyl‐CoA levels are likely too low to generate detectable quantities of acetate from this pathway. Cells might also metabolize pyruvate/acetyl‐CoA through the glyoxylate shunt, which is induced when glucose is absent [45, 46]. Whereas acetate formation would permit ATP generation, the TCA cycle or the glyoxylate shunt would allow cells to generate precursor molecules as well as NADH. These pathways providing a limited acetyl‐CoA supply to generate energy enable the complete conversion of pyruvate to acetoin. When acetyl‐CoA metabolism is essentially blocked, however, as is the case with MEC1342/44_ediss, the conversion of pyruvate to acetoin cannot be sustained in the absence of glucose.

During controlled batch processes, less than 2 g/L acetolactate accumulated during exponential growth, and this intermediate was metabolized by the end of the process. A likely explanation for this observation is that in vivo ALS activity is greater than that of ALDC. Of these two enzymes, only kinetic parameters for ALDC from *E. cloacae* ssp. *dissolvens* are available: the K_M_ is 12.2 mM, and k_cat_ is 0.96 s^−1^ [47] while known ALS enzymes have a turnover number 100 times greater [48]. Even without kinetic parameters for the specific ALS used, the low affinity turnover rate of ALDC, taken together with the observed accumulation of acetolactate, support the hypothesis that ALDC is the rate limiting step for acetoin formation. About 5 g/L 2R,3R‐butanediol was also detected in the prolonged fed‐batch process. Although a heterologous butanediol dehydrogenase was not expressed, the presence of this biochemical is likely due to promiscuous activity of endogenous dehydrogenases as previously reported in *E. coli* [29, 49].

Competition between central metabolism and product pathways for metabolites directly impacts the metabolic flux toward a product of interest. Central metabolic enzymes typically have high turnover and catalytic efficiencies compared with enzymes of secondary metabolism [50]. Thus, an introduced pathway must invariably compete directly with central metabolism. Common strategies to increase flux through product pathways include gene deletion and the overexpression of pathway genes. In this study, implementation of these two strategies alone in batch culture resulted in 2.7 g/L acetoin at a yield of 0.07 g/g (MEC1319/44_ediss, Figure 4A), much lower than the maximum theoretical yield of acetoin from glucose of 0.489 g/g. Wild‐type PDH has a K_M_ of 260 µM and a k_cat_ of 38 s^−1^ [38]. The kinetic parameters of ALS from *E. cloacae* ssp. *dissolvens* have not been characterized, though ALS from *Bacillus subtilis* has a K_M_ of 13,600 µM and a k_cat_ of 121 s^−1^ [48]. The intracellular pyruvate concentration when *E. coli* is growing maximally is about 5000 µM [51, 52]. Using Michaelis‐Menten kinetics and these parameters as benchmarks, the wild‐type PDH is operating at near its maximum reaction rate (i.e., about 36 s^−1^), while ALS is at 25% of its maximum (33 s^−1^). However, increased ALS expression would tend to lower intracellular pyruvate concentration, which would reduce the reaction rate of ALS more than for PDH because of the much greater K_M_ of ALS, making ALS even less competitive. In contrast, a variant strain with reduced PDH activity would lower the reaction rate for the conversion of pyruvate to acetyl‐CoA, and would also tend to increase intracellular pyruvate concentration, both benefiting acetoin formation. Although these calculations do not consider the complex phenomena which influence enzyme kinetics in vivo, including expression level, protein turnover and the presence of inhibitors, this analysis serves to illustrate the benefit of reduced PDH flux via the modification of the native enzyme.

An important result is that a severe restriction in PDH flux does not correlate to high acetoin yield and productivity when the heterologous pathway is introduced into an *aceE* variant strain. Of the four strains compared in batch culture, MEC1332/44_ediss (AceE[H106V]) achieved the greatest acetoin productivity and yield (Figure 4C) even though MEC1342/44_ediss (AceE[H106M;E401A]) directed more carbon to pyruvate and acetoin (Figure 4D). MEC1332/44_ediss also maintained this performance in repeated batch processes, which permitted prolonged acetoin production by the periodic addition of glucose. Thus, the optimal restriction in PDH flux appears to be contextual, and likely depends on the particular kinetic parameters of the introduced pathway, as well as their expression level and the energy demands associated with plasmid and protein maintenance. However, this trend was not observed in results from the initial screening in shake flasks (Figures 2 and 3). The decreased performance of MEC1342/44_ediss (AceE[H106M;E401A]) under controlled batch conditions is evident when glucose is depleted and the pyruvate concentration is relatively high, a circumstance that would not occur during screening in shake flasks with relatively low glucose concentrations (5 g/L). The difference in strain performance between shake flasks and controlled reactors highlights the importance of assessing strains in controlled reactors as results from initial screening experiments are not always consistent when a process is scaled‐up.

Targeted modulation of PDH to increase the yield of acetoin has, to the best of our knowledge, not been explored. Previously, acetoin generation with a pyruvate‐producing strain of *E. coli*, containing deletions in *ldhA*, *poxB, ppsA*, and *aceEF* among others, has been studied using the rationale that the increased availability of pyruvate will lead to increased acetoin formation [29]. However, the strain only produced 0.87 g/L acetoin using complex medium (LB) with 10 g/L glucose, a result similar to our current results for MEC1319/44_ediss, which led to 2.7 g/L acetoin from 40 g/L glucose as the sole carbon source. Restricting flux through PDH benefits pyruvate and acetoin formation. However, simply eliminating PDH activity and providing for growth with acetate supplementation is not an optimal strategy for producing pyruvate‐derived compounds.

Expression of an enzyme variant with modified kinetic parameters is just one method to modulate the flux at a key branch point in central metabolism, and this approach complements the modification of the promoter for the gene(s) coding for the same enzyme. The use of inducible or characterized, constitutive promoters are well suited for monocistronic genes or operons lacking regulatory networks. For example, the native *aceE* promoter was replaced with a weaker promoter to decrease the expression of *aceE*, resulting in improved L‐valine production in *Corynebacterium glutamicum* [53]. In *C. glutamicum*, *aceE* is monocistronic and not significantly regulated [54]. In *E. coli*, *aceE* is part of an operon where the three components of the PDH (*aceE*, *aceF*, and *lpdA*) are transcribed with *pdhR*, whose gene product acts as a pyruvate‐sensitive global repressor for the *pdhR‐aceEF‐lpdA* operon as well as other genes involved in cell replication, motility, and respiration [55, 56, 57]. Moreover, though the *pdhR* promoter dominates *aceEF* expression, promoter sequences upstream of *aceEF*, independent from PdhR regulation, can also drive *aceEF* expression [58, 59, 60]. Thus, expression from P*pdhrR* and P*aceE* must both be reduced to modulate PDH activity in *E. coli* effectively [18]. Complex transcriptional regulation and redundancy of promoters can pose a challenge when engineering weak promoters to decrease carbon flux through a branchpoint. As an alternative to decreasing the expression of a gene transcribed within a complex operon, the use of enzyme variants with decreased activity represents a simple strategy to accomplish a similar goal of reducing flux.

The titer of two pyruvate‐derived products, acetoin and 2R,3R‐butanediol, reached 42.5 g/L with a yield of 0.34 g/g at a productivity of 1.0 g/L·h in a medium with glucose as the sole carbon source, corresponding to an effective concentration of 59 g/L considering the dilution due to the glucose feed. Among the strains examined in the context of acetoin formation, the *aceE* ^[H106V]^ allele allowed the optimal carbon distribution at the pyruvate node for acetoin production, offering the ideal balance for cells between metabolizing pyruvate through the PDH to allow growth and partitioning a portion of the pyruvate for acetoin production. A potential advantage of using PDH variants is that they can maximize the effectiveness of a kinetically slow, introduced pathway by directly partitioning the precursor metabolite between growth and the introduced pathway, allowing for a fine tuning of metabolism aligned with cellular demands.

This approach of modifying native enzyme activity could readily be combined with existing strategies such as promoter engineering, optimization in introduced pathway genes or other chromosomal modifications.

## CONFLICT OF INTEREST

The authors have declared no conflict of interest.

## Supporting information

Supplemental Material
