# Supplementary material for: Escherichia coli aceE variants coding pyruvate dehydrogenase improve the generation of pyruvate‐derived acetoin
Source: Eng Life Sci. 2023 Jan 31;23(3):e2200054. doi: 10.1002/elsc.202200054 (PMC9978916; doi:10.1002/elsc.202200054)
Supplement: Supplementary file 1 — Supplemental Material [file ELSC-23-e2200054-s001.docx]

**Supplemental Material**

**Improving the generation of pyruvate-derived acetoin by *Escherichia coli aceE* variants coding pyruvate dehydrogenase**

W. Chris Moxley, Rachel E. Brown, Mark A. Eiteman

**Table S1.** Plasmids used in this study.

| Name | Relevant characteristics | Description | Source |
| --- | --- | --- | --- |
| pKD4 | Amp^R^, Kan^R^; R6K ori | Source of Kan^R^ cassette | Datsenko et al. 2000 |
| pKD46 | Amp^R^; pSC101 ori (ts); *araBAD* promoter for λ Red genes | λ Red helper plasmid | Datsenko et al. 2000 |
| pCP20 | Amp^R^, Cam^R^; pSC101 ori (ts) | Expression of FLP recombinase | Datsenko et al. 2000 |
| pCM02 | Amp^R^; pUC ori | *aceE* template for EP-PCR | Moxley and Eiteman, 2021 |
| 445_ediss | Kan^R^; pUC ori | Expression of *budB*, *budA*, and *budC* | Erian et al., 2018 |
| 44_ediss | Kan^R^; pUC ori | Expression of *budB* and *budC* | This study |

**Table S2.** Primers used in this study.

| Name | Description | Sequence 5'-3' |
| --- | --- | --- |
| MEP166 | ldhA_F | TTAAGCATTCAATACGGGTATTGTG |
| MEP167 | ldhA_R | GTCATTACTTACACATCCCGCCATC |
| MEP168 | aceE_F | TGAGCGTTCTCTGCGTCGTCTGGA G |
| MEP169 | aceE_R | ATCGCCAACAGAGACTTTGATCTC |
| MEP288 | poxB_F | CCGGTTGTCGCTGCCTGC |
| MEP289 | poxB_R | TTCAAACAGATAGTTATGCGCGGCC |
| MEP291 | ppsA_R | CGTTTAGGTGAACGATCATGCGC |
| MEP418 | ace-seq-1 | GAAATATCTGGAACACCGTGG |
| MEP419 | ace-seq-2 | CCAAAGGCAAAGCGACAG |
| MEP420 | ace-seq-3 | CTTACTATAAAGAAGACGAGAAAGGTC |
| MEP503 | KD4-HA-poxB-F | GATGAACTAAACTTGTTACCGTTATCACATTCAGGAGATGGAGAACCATGGTGTAGGCTGGAGCTGCTTC |
| MEP504 | KD4-HA-poxB-R | CCTTATTATGACGGGAAATGCCACCCTTTTTACCTTAGCCAGTTCGTTTTCATATGAATATCCTCCTTA |
| MEP579 | pKD4-HA-Cpps-F | AGAAATGTGTTTCTCAAACCGTTCATTTATCACAAAAGGATTGTTCGATGGTGTAGGCTGGAGCTGCTTC |
| MEP671 | KD4_RB_ldhA_F | TATTTTTAGTAGCTTAAATGTGATTCAACATCACTGGAGAAAGTCTTATGGTGTAGGCTGGAGCTGCTTC |
| MEP795 | pKD4-REB-aceE-F | ACAGGTTCCAGAAAACTCAACGTTATTAGATAGATAAGGAATAACCCATGGTGTAGGCTGGAGCTGCTTC |
| MEP796 | pKD4-REB-aceE-R | GATTTCGATAGCCATTATTCTTTTACCTCTTACGCCAGACGCGGGTTAACCATATGAATATCCTCCTTAG |
| MEP848 | aceE-EP-F | ATGTCAGAACGTTTCCCAAATGACGTGGATCCGATCGAAACTCGCGACTGGCTCCAGGCG |
| MEP849 | cmsB-HA-aceE EP-F | AGTATCTGATCGACCAACTGCTTGCTGAAGCCCGCAAAGGCGGTGTAAACTGTGACGGAAGATCACTTCG |
| MEP850 | cmsB-HA-aceE EP-R | GCCCAGCGCCGCAACCACGACATAAGAAGCATCAACTTCGAAGTGGTGACGCTGTCCATATGCACAGATG |
| MEP851 | aceE-EP-R | TTACGCCAGACGCGGGTTAACTTTATCTGCATCGATGTTGAATTTGGCGATTGCGTCAGC |
| MEP920 | ediss budC F | CTGGCCCGGCGAGGTATGAAGTCTCATCGTTGTAGTCGGCGATAG |
| MEP921 | ediss budC R | TTCGTATCTCGCCGGGCCAGATTCCGACTACATGACCGGCCAGTC |
| MEP1010 | KD4-R-W-ldhA | CTCCCCTGGAATGCAGGGGAGCGGCAAGATTAAACCAGTTCGTTCGGGCACATATGAATATCCTCCTTAG |
| MEP1011 | ppsA-F-W | CGCACAGAAGCGTAGGACGTAATG |
| MEP1012 | KD4-R-W-ppsA | CGACTGAACGCCGCCGGGGATTTATTTTATTTCTTCAGTTCAGCCAGCATATGAATATCCTCCTTAG |
|  |  |  |
|  |  |  |
|  |  |  |
|  |  |  |
|  |  |  |
|  |  |  |

**References**

Datsenko KA, Wanner BL. 2000. One-step inactivation of chromosomal genes in *Escherichia coli* K-12 using PCR products. *Proc Natl Acad Sci* 97:6640–6645.

Erian AM, Gibish M, Pflügl S. 2018. Engineered *E. coli* W enables efficient 2,3-butanediol production from glucose and sugar beet molasses using defined minimal medium as economic basis. *Microb Cell Fact* 17, 190.

Moxley WC, Eiteman MA. 2021. Pyruvate production by *Escherichia coli* by use of pyruvate dehydrogenase variants. *Appl Environ Microbiol* 87:e00487-21.
